# A Systematic Approach to Managing Acute Visual Loss: A Comprehensive Review of Literature

**DOI:** 10.7759/cureus.102280

**Published:** 2026-01-25

**Authors:** Fatima Hajj, Padraig Totten, Doria Stephanie Chavez, Diana Carolina Cortés Jaimes, Diti Patel, Gabriel Andres Soria Behr, Gilberto González, Avin Khushiramani

**Affiliations:** 1 Medicine, Lebanese University Faculty of Medicine, Beirut, LBN; 2 Internal Medicine, Northern Ireland Medical and Dental Training Agency, Belfast, GBR; 3 General Medicine, Universidad de Guadalajara Centro Universitario de Ciencias de la Salud, Guadalajara, MEX; 4 Medicine, Pontificia Universidad Javeriana, Bogotá, COL; 5 Ophthalmology, Universidad Industrial de Santander, Floridablanca, COL; 6 Epidemiology, Universidad Autónoma de Bucaramanga, Bucaramanga, COL; 7 Ophthalmology, Baroda Medical College, Vadodara, IND; 8 Internal Medicine, University of Guayaquil, Guayaquil, ECU; 9 Medicine, Tecnológico de Monterrey, Campus Monterrey, Monterrey, MEX; 10 Ophthalmology, Maharashtra Institute of Medical Education and Research (MIMER) Medical College, Talegaon, IND

**Keywords:** acute vision loss, diagnosis, emergency department, management, optic neuropathy, red flags, retina

## Abstract

Acute vision loss is a clinical emergency that needs to be diagnosed promptly and managed early in order to prevent irreversible vision loss. This review provides a comprehensive synthesis of literature regarding differential diagnosis, diagnostic approaches, management strategies, and red flags of acute vision loss. A systematic review of relevant articles was conducted using databases such as PubMed and Scopus. The findings highlight that the rapid differentiation among various etiologies of acute vision loss through detailed history-taking, thorough ophthalmic examination, and other investigations is crucial for timely intervention. The early diagnosis of the underlying etiology is essential for initiating appropriate treatment and preserving the vision. Despite improvements in diagnostic techniques, delayed diagnosis is still a major challenge. This review outlines a systematic approach to acute vision loss and provides future research directions to enhance early detection and patient outcomes.

## Introduction and background

In the United States, virtually every person who reaches average life expectancy will experience some form of vision loss. Estimates indicate that up to 90 million adults over age 40 have vision problems, ranging from refractive error to age-related macular degeneration [1]. While chronic vision impairment often follows a progressive, predictable course, acute visual loss is distinguished by its sudden onset, typically occurring within seconds to days, and its status as a clinical emergency that necessitates immediate triage to differentiate reversible pathology from permanent disability. This highlights a substantial population burden in which acute visual loss episodes represent a common and clinically urgent manifestation of overall ocular epidemiology.

At the population level, acute visual loss imposes a substantial burden on healthcare systems. In Wales, the overall incidence is 65.4 episodes per 1,000 people per year, with only 0.9% resulting in general practitioner (GP)-prescribed medication and 0.6% leading to emergency attendance. Optometrists manage the majority of cases (51.8%), compared with 0.6% each for pharmacy and emergency services [2]. While these data reflect a UK-based system where community optometry serves as a robust primary gatekeeper, the findings highlight a universal pressure on healthcare resources. In systems without integrated community eye care, such as many regions in North America, these acute episodes often shift more heavily toward emergency departments (EDs), further intensifying the need for rapid diagnostic protocols. These patterns emphasize the need for targeted workforce planning and public awareness strategies to encourage the appropriate use of available services, particularly among socially disadvantaged groups who experience a higher burden of episodes.

The early recognition of acute vision loss in the emergency department is essential because it facilitates immediate neuroimaging and timely, condition-specific interventions, such as thrombolysis for stroke, psychiatric care for psychogenic blindness, or steroid therapy for optic neuritis, thereby preventing irreversible visual and neurological damage [3]. Because isolated visual symptoms can be the only sign of an occipital infarction, prompt diagnosis enables timely thrombolytic therapy, significantly improving recovery and reducing morbidity and mortality. Conversely, delayed presentation underscores the need for rapid visual and neurological assessment to identify cortical blindness and initiate appropriate stroke management [4].

## Review

Classification of acute vision loss

There are several classifications for acute visual loss. It may be binocular (the loss of vision in both eyes) or monocular (the loss of vision in one eye). Monocular complaints indicate illness processes that occur before the chiasm. Binocular problems can affect any of the chiasm's more posterior projections or the chiasm itself. The loss of vision may be painless, or it may be associated with pain. Acute angle-closure glaucoma usually manifests as a painful loss of vision [5]. Transient vision loss, often referred to as amaurosis fugax, is characterized by a brief, temporary deficit in vision, typically lasting from a few seconds to several minutes and rarely exceeding 24 hours, that resolves spontaneously. However, it is critical to emphasize that spontaneous resolution does not imply a benign prognosis; rather, these episodes often serve as a sentinel warning for high-risk vascular etiologies, such as carotid artery stenosis or impending stroke, necessitating an urgent systemic evaluation even after vision has returned to baseline [5-7]. Patients with amaurosis fugax will typically appear with either monocular or binocular temporary vision loss. Transient vision loss may result from vascular causes such as carotid occlusion, thromboembolism, giant cell arteritis (GCA), ocular ischemic syndrome, or cardioembolic sources or from benign causes such as migraine or retinal vasospasm [6]. Although there are several reasons for it, temporary retinal ischemia is the most common cause [7]. Sudden vision loss is an abrupt loss of eyesight. The most frequent clinical disorders that result in sudden vision loss include retinal detachment, papilledema, vitreous hemorrhage, ischemic optic neuropathy, optic neuritis, and central retinal artery/vein occlusion [8]. It usually occurs due to the acute disruption of retinal or optic nerve function, most commonly from ischemic or inflammatory injury. In retinal vascular occlusion, hypoperfusion rapidly leads to photoreceptor damage within minutes, whereas in optic neuritis, immune-mediated demyelination causes acute visual decline, often accompanied by color desaturation. Prompt recognition and immediate intervention, such as high-dose corticosteroids for optic neuritis or ocular massage and thrombolysis for central retinal artery occlusion (CRAO), are crucial to prevent permanent vision loss [9].

Differential diagnosis

Acute vision loss is a medical emergency that must be assessed immediately. Given that acute vision loss may arise from a wide array of etiologies, determining the underlying cause is crucial for timely management. The clinical evaluation begins by categorizing the type of visual loss according to three primary diagnostic pillars: whether it is associated with pain or is painless, monocular (affecting one eye) or binocular (affecting both eyes), and transient or constant. The causes of acute loss of vision can be broadly categorized into anterior segment disorders, retinal diseases, optic nerve pathologies, and neurovascular diseases. A systematic approach based on a detailed ocular history and focused examination helps narrow the differential diagnosis and determines the urgency of referral for ophthalmologic assessment [10].

The first priority is to identify whether the vision loss is monocular or binocular, as this distinction provides a critical anatomical clue. Monocular deficits typically implicate ocular, optic nerve, or pre-chiasmal pathology. Conversely, bilateral or field-crossing defects raise immediate concern for chiasmal, retro-chiasmal, or central nervous system (CNS) etiologies [11]. Once laterality is established, the clinician must determine whether the onset is painful or painless and whether the loss is transient or persistent. Ocular pain and photophobia are usually present in corneal pathologies, whereas pain specifically elicited by ocular movement is highly suggestive of retrobulbar optic neuritis. Temporary vision loss lasting less than 24 hours may be due to media-related issues, vascular insufficiency, or optic disc and neurological issues, presenting as blurry vision, dimming, or a reduced visual field [10].

A detailed temporal profile, whether the loss evolved within seconds, as seen in vascular occlusion, or over hours to days, as in optic neuritis or other inflammatory etiologies, further refines lesion localization and guides the urgency of intervention. A comprehensive bedside evaluation should include the assessment of visual acuity, color vision, pupillary reflexes, visual fields, and a fundus examination to identify retinal or optic nerve pathology early. Incorporating these structured clinical steps ensures the systematic localization of the lesion and timely differentiation between ocular, neuro-ophthalmic, and systemic causes of acute vision loss [12]. Among the painless causes, retinal vascular occlusions such as central retinal artery occlusion (CRAO) and central retinal vein occlusion (CRVO) are major considerations. CRAO is an ophthalmic emergency that typically manifests as a sudden, painless, and severe loss of vision in the involved eye with a characteristic fundoscopic finding of a cherry-red spot [13]. In cases of CRVO, vision loss is painless and unilateral, with reduced visual acuity reflecting the degree of venous obstruction. Fundoscopic findings in CRVO often show superficial and deep intraretinal hemorrhages in all four quadrants of the retina associated with variable degrees of retinal venous engorgement and tortuosity, optic disc swelling, cotton wool spots, and cystoid macular edema [14].

Retinal detachment should also be suspected when a patient presents with symptoms of flashes of light, known as retinal photopsia, visual floaters, and peripheral, typically progressive, visual field loss [15]. Ischemic optic neuropathy presents with sudden, unilateral, painless vision loss, often associated with systemic hypotension, and may show a relative afferent pupillary defect (RAPD) and visual field loss [16]. The optic nerve-related causes include idiopathic demyelinating optic neuritis, which is characterized by painful eye movements and rapid vision loss peaking in 7-10 days, often with mild disc swelling. Non-arteritic anterior ischemic optic neuropathy (NAION) manifests as painless, sectoral disc edema with altitudinal field loss, commonly in patients over 50 years of age with vascular risk factors. In contrast, arteritic anterior ischemic optic neuropathy (AAION) due to giant cell arteritis (GCA) presents as chalky white disc edema accompanied by systemic symptoms and an elevated erythrocyte sedimentation rate (ESR) or C-reactive protein (CRP). Less common but important differentials include neuromyelitis optica, which is often bilateral and severe, inflammatory or infectious optic neuropathies such as syphilis or Lyme disease, and hereditary conditions such as Leber hereditary optic neuropathy (LHON). Finally, compressive or neoplastic lesions may mimic optic neuritis on presentation and must be considered in the clinical workup [17].

Diagnostic tools

Acute vision loss is a diagnostic challenge that requires immediate evaluation to distinguish among retinal, vascular, optic nerve, and neuro-ophthalmic etiologies. The first-line intervention remains a detailed clinical history and fundus examination to look for hallmark signs such as cherry-red spot, optic disc edema, vessel narrowing, or the presence of hemorrhage.

Acute visual loss should be evaluated with a rapid, systematic ophthalmic examination that includes the measurement of distance visual acuity, a pinhole test to exclude refractive error, and the assessment of color vision using red desaturation or pseudoisochromatic plates [18]. In the acute visual loss setting, a slit lamp examination is indispensable for evaluating anterior segment pathology, allowing the direct visualization of corneal clarity, the detection of epithelial defects with fluorescein staining, and the identification of findings such as hyphema, ulceration, or inflammatory infiltrates that can account for the sudden visual deficit [19].

Fundus fluorescein angiography (FFA) allows the examination of the vasculature of the retina while also highlighting dynamic effects produced by blood vessel leakage from endothelial injury, inflammation, neovascularization, or elevated intracranial pressure, as well as delays in vascular filling in various structures [20]. In case of central retinal artery occlusion (CRAO), FFA is often performed at the clinician's discretion to record the arm-retina time, which helps estimate the location and degree of occlusion and final visual outcome. Additionally, FFA is useful in ruling out partial or total ophthalmic artery occlusion [21] and remains a pivotal diagnostic tool for visualizing retinal and choroidal blood flow, highlighting vascular leakage and neovascularization, and thereby facilitates the rapid identification of conditions such as diabetic retinopathy and macular degeneration that underlie sudden visual impairment [22].

Optical coherence tomography (OCT) is an imaging technique that provides a three-dimensional view of the retina within seconds, allowing clinicians to view each retinal layer and optic nerve head in real time [23]. OCT helps to distinguish the sequelae of retinal artery blockage from those of primary optic neuropathy [24].

Acute visual loss after cerebral angiography, most often manifesting as transient cortical blindness, shows characteristic but frequently subtle imaging findings on CT, MRI, and CT angiography. Non-contrast CT may reveal a gyriform hyperattenuation in the parieto-occipital cortex, interpreted as contrast extravasation from blood-brain barrier disruption, yet many scans are completely normal. Follow-up MRI typically demonstrates bilateral occipital cortical hyperintensity on T2-weighted and fluid-attenuated inversion recovery (FLAIR) sequences with isointense T1 signal and without restricted diffusion, reflecting reversible vasogenic edema rather than infarction. Diffusion-weighted imaging is consistently negative, helping exclude embolic ischemia. CT angiography usually confirms intact arterial patency and excludes occlusive disease while implicating the intra-arterial contrast medium as the pathogenic agent. These imaging abnormalities generally resolve within days and parallel the rapid clinical recovery observed in most patients [25].

The timely integration of these diagnostic modalities not only aids in accurately identifying the underlying cause of acute vision loss but also guides prompt and targeted management, ultimately improving visual prognosis and preventing irreversible ocular damage.

Red flags and referral criteria

The early identification of red flags in patients presenting with acute visual loss is essential for prompt diagnosis and treatment to prevent irreversible blindness. Clinical red flags include sudden, painless loss of vision, severe eye pain, headache, photophobia, and visual field defects, as well as associated neurological symptoms such as diplopia (double vision), focal weakness, or altered consciousness. Crucial physical examination findings that serve as warning signs include a relative afferent pupillary defect (RAPD), objective visual field deficits, and optic disc swelling, all of which are highly suggestive of an underlying optic neuropathy (Table 1) [17].

**Table 1:** Clinical Red Flag Checklist and Referral Criteria for Acute Visual Loss RAPD, relative afferent pupillary defect; CRAO, central retinal artery occlusion; CRVO, central retinal vein occlusion; CNS, central nervous system

Clinical Finding	Potential Etiology	Referral Urgency
Sudden, painless vision loss	Vascular occlusion (CRAO/CRVO) or retinal detachment	Emergent (immediate)
Severe pain with nausea/vomiting	Acute angle-closure glaucoma	Emergent (immediate)
Scalp tenderness/jaw claudication	Giant cell arteritis (GCA)	Emergent (immediate)
Positive RAPD or disc swelling	Optic neuropathy/ischemic optic neuropathy	Urgent (same-day)
Bilateral or atypical optic neuritis	CNS inflammatory disease/neuromyelitis optica	Urgent (24-48 hours)
Associated focal neurodeficits	Stroke or intracranial mass	Emergent (immediate)

In older individuals between 70 and 79 years of age, clinical features such as headache, jaw or limb claudication, visual loss, scalp tenderness, or stroke are critical red flags for giant cell arteritis (GCA). These symptoms necessitate the immediate initiation of high-dose glucocorticoid therapy and same-day ophthalmology consultation if the patient reports vision loss or double vision [26]. Acute visual loss in GCA represents a critical emergency, as irreversible optic nerve damage can occur within hours of arterial occlusion. The underlying mechanism is usually arteritic anterior ischemic optic neuropathy (AAION) due to the occlusion of the posterior ciliary arteries supplying the optic nerve head. The prompt recognition of these systemic symptoms is vital, as early intervention can restore perfusion before infarction becomes permanent [27].

Additionally, bilateral vision loss, an atypical age of onset (greater than 50 or less than 12 years), the absence of pain, and a lack of recovery are atypical features of optic neuritis that signal the need for urgent neuroimaging and further systemic investigations [28]. The presence of systemic symptoms, such as sinusitis or sicca symptoms (dry eyes and mouth), and unusual neurological signs such as acute transverse myelitis, area postrema syndrome (unexplained hiccups or nausea), or ataxia in cases of atypical optic neuritis indicate central nervous system (CNS) involvement or systemic inflammatory etiologies. These findings should trigger an urgent multidisciplinary referral to neurology and ophthalmology. Adhering to these red flag recognition and referral criteria is essential for ensuring timely diagnosis and optimal visual outcomes in patients presenting with acute visual loss [29].

Management principles

The management of acute vision loss requires a systematic, timely, multidisciplinary approach to preserve vision and prevent irreversible vision loss. The initial evaluation includes prompt recognition and accurate diagnosis through detailed history-taking (onset, duration, unilateral versus bilateral involvement, and associated symptoms such as pain, flashes, floaters, headache, or jaw pain) and thorough ophthalmic examination (visual acuity, intraocular pressure, pupillary responses, and fundoscopy) to help localize the lesion [30]. Because certain causes, such as central retinal artery occlusion (CRAO), represent an acute ischemic stroke of the retina, immediate triage to an emergency department is essential, and delay for outpatient workup should be avoided [31]. The management of acute vision loss should begin with a comprehensive ocular examination, including external inspection, visual acuity and visual field testing, pupillary assessment, extraocular movement evaluation, slit lamp biomicroscopy, ophthalmoscopy, and intraocular pressure measurement, followed by point-of-care ultrasound to rapidly identify or exclude posterior segment emergencies such as retinal detachment, vitreous hemorrhage, or cataract, thereby streamlining disposition and enabling prompt ophthalmology consultation [30]. Optical coherence tomography can rapidly reveal inner-retinal thickening from edema in CRAO, and fluorescein angiography may show delayed or absent perfusion if needed, aiding in the confirmation of retinal ischemia [31].

The effective management of acute retinal necrosis is essential to curb rapid vision loss; the current standard combines high-dose systemic antiviral therapy with adjunctive intravitreal antivirals and corticosteroids and may include prophylactic laser retinopexy to halt disease progression and protect the fellow eye. Although, even with aggressive treatment, visual outcomes remain modest and retinal-detachment rates are high, the early initiation of this regimen has been shown to prevent contralateral involvement and offers the best chance for preserving visual function [32]. Similar to other retinal ischemic emergencies such as central retinal artery occlusion (CRAO), the timely diagnosis and initiation of targeted therapy are crucial, as delays can result in irreversible retinal damage. In both conditions, coordinated multidisciplinary management, including urgent ophthalmology involvement and systemic evaluation, is vital to address underlying vascular or infectious etiologies and to optimize visual prognosis [31].

Acute retinal artery occlusion, a leading cause of sudden vision loss, must be treated as an ophthalmic emergency, prompting immediate referral to an emergency department with stroke expertise and a comprehensive systemic workup, including cardiac and carotid imaging, to identify underlying embolic or inflammatory sources [33]. Early recognition and rapid coordination between ophthalmology and stroke teams are vital to optimize outcomes. The timely initiation of reperfusion strategies where appropriate, followed by targeted secondary-prevention measures such as antiplatelet therapy and vascular risk-factor control, remains essential to reduce the likelihood of recurrent ischemic events and preserve residual visual function [31]. The therapeutic goal is to restore retinal blood flow within the narrow window of irreversible retinal injury, approximately 240 minutes after onset. The timely initiation of reperfusion therapy, such as ocular massage, thrombolysis (intravenous within ≤4.5 hours or intra-arterial within ≤12 hours), hyperbaric oxygen, or intraocular pressure-lowering measures, may offer potential vision-saving benefits. Simultaneously, systemic vascular risk factors should be addressed through antithrombotic therapy, statin therapy, blood pressure management, and anticoagulation if atrial fibrillation is present to reduce the high risk of subsequent cerebrovascular events [34].

Acute retinal arterial ischemia, whether presenting as transient monocular visual loss, branch retinal artery occlusion, or central retinal artery occlusion, requires urgent brain MRI with diffusion-weighted imaging and vascular studies, along with immediate referral to a stroke center or rapid-access transient ischemic attack (TIA) clinic. Comprehensive workup should include basic laboratory investigations, electrocardiography, cardiac monitoring if available, noninvasive carotid and cranial vessel imaging, and echocardiography to identify treatable embolic sources [35]. Time to treatment has been identified as a critical prognostic factor in retinal ischemia management, with the early initiation of thrombolytic or antithrombotic therapy significantly improving visual outcomes when administered within the first few hours of symptom onset [36]. Early recognition and referral enable the initiation of secondary prevention strategies, including dual antiplatelet therapy, statins, and expedited carotid revascularization when indicated, thereby minimizing the risk of recurrent stroke and preserving remaining visual function [35]. Furthermore, ongoing research highlights the need for standardized retinal perfusion metrics and evidence-based therapeutic windows to optimize individualized treatment protocols for acute retinal ischemia [36].

To sum up, the management of acute vision loss hinges on rapid recognition, precise diagnosis, and timely multidisciplinary intervention to prevent irreversible visual impairment. The early initiation of targeted therapy; close collaboration between ophthalmologists, neurologists, and emergency physicians; and prompt implementation of systemic preventive measures remain the cornerstones for optimizing visual and neurological outcomes.

Challenges in emergency settings

The emergency management of acute vision loss poses significant challenges due to the time-sensitive nature of many underlying etiologies and the broad differential diagnosis that covers ocular, neurological, and systemic causes. Physicians must rapidly identify the anatomical level of the defect, determine which time-critical interventions are required, and arrange appropriate specialty consultation while often working with limited high-quality evidence, as most data are derived from case series or expert opinion. A focused history that elicits onset, laterality, pain, and associated neurological symptoms is the first diagnostic step, yet patients may mask diplopia as "blurred" vision or fail to notice unilateral loss [37]. The physical examination must be systematic, visual acuity, visual fields, pupillary testing (including the swinging-flashlight test for an afferent defect), extraocular movements, and anterior and posterior segment evaluation, because subtle findings, such as a painless homonymous hemianopia, can uncover life-threatening CNS pathology. Red flag features such as sudden painless loss, photophobia, eye pain, decreased acuity, or systemic symptoms (fever and myalgias) should prompt urgent imaging and laboratory workup (ESR/CRP, toxicology, neuroimaging). Finally, some bedside tools, such as digital tonometry and prolonged topical anesthetic use, are unreliable and can mislead the clinician, underscoring the need for careful technique and early ophthalmology involvement [37].

In community-based emergency departments (EDs), physicians often lack familiarity with neuro-ophthalmic disorders, and many EDs have limited or no on-site ophthalmology coverage, leaving clinicians without timely specialist input. Tele-neurology consultations frequently lack posterior segment visualization, and radiology protocols are not standardized, resulting in inappropriate or insufficient imaging. Laboratory workups for conditions such as giant cell arteritis are frequently omitted, and essential bedside tools, non-mydriatic fundus cameras and external ocular photographs, are often unavailable, impeding accurate diagnosis. These gaps in knowledge, equipment, and communication can delay critical interventions such as high-dose corticosteroids, leading to irreversible vision loss or even death. Bridging these barriers through image capture, Health Insurance Portability and Accountability Act of 1996 (HIPAA)-compliant messaging, and clear clinical guidelines is essential to improve outcomes for patients with acute visual loss in the ED [38].

Emergency settings also face systemic and resource-related barriers. Inadequate healthcare infrastructure limits the availability of equipped eye care facilities, while a severe shortage and uneven distribution of trained ophthalmologists, optometrists, and eye care nurses restrict timely specialist intervention. Socioeconomic constraints and remote locations further delay presentation, as patients in rural or low-income areas often face long travel distances, high transportation costs, and limited affordability of services, leading to delayed diagnosis and treatment [39].

When tele-stroke services are engaged, neurologists often lack essential ophthalmic information, leading to costly or inappropriate testing and delayed diagnosis of non-stroke eye-related conditions. In hospitals without on-site ophthalmology, an emergent ophthalmic examination is rarely feasible; patients with isolated painless monocular loss require prompt fundoscopic evaluation, yet many centers must rely on limited ultrasound or defer to outpatient follow-up. Even when ophthalmology is available, performing a reliable fundus examination is hampered by the need for specialized equipment, technical skill, and interpretive expertise, capabilities that most emergency physicians and residents have not mastered in over two decades of medical education. Consequently, only a small fraction of eligible patients receive a fundus examination, and missed findings can alter management decisions. Compounding these issues, a substantial proportion of small and rural hospitals lack on-call ophthalmologists, with staffing gaps reported in 73% of surveyed facilities. Although emerging tele-ophthalmology tools, such as smartphone adapters and non-mydriatic cameras, offer potential solutions, their integration into existing tele-stroke workflows remains limited, leaving a critical gap in the rapid triage of acute visual loss [40].

Overall, these challenges highlight the need for improved emergency training, access to ophthalmic resources, and streamlined interdisciplinary collaboration to prevent irreversible vision loss.

Future directions

Future strategies in the management of acute vision loss must emphasize early detection, rapid triage, and efficient interdisciplinary care to improve clinical outcomes. Advances in tele-ophthalmology combined with artificial intelligence (AI)-driven diagnostic tools offer a promising solution for resource-limited and emergency settings, allowing remote fundus image interpretation and automated pattern recognition that empower nonspecialist clinicians to make timely diagnoses [41]. Integrating point-of-care diagnostics such as portable OCT and handheld fundus cameras, to capture high-resolution retinal images at the bedside or in primary-care settings, enables rapid remote ophthalmology triage and timely referrals [42]. Moreover, recent deep learning systems have demonstrated high accuracy in detecting hyperacute central retinal artery occlusion within the critical 4.5-hour therapeutic window, outperforming clinical neurologists in some datasets [43]. Another promising direction is the AI-driven quantification of retinal vascular parameters in uveitis, particularly reduced vein tortuosity observed in ocular tuberculosis (TB), that offers a novel biomarker to differentiate TB-related uveitis from other uveitis causes (QuantiFERON-TB Gold {QFT}-positive uveitis and ocular toxoplasmosis), thereby enhancing diagnostic precision and monitoring treatment response [44].

In addition to advances in diagnostics, innovations in surgical management such as microincision pars plana vitrectomy (PPV) represent another key frontier. Recent evidence supports the superiority of the newer 27-gauge (27G) platform over the traditional 25-gauge (25G) system, offering a safer, suture-less, and equally efficient approach in the time-critical setting of acute vision loss. However, small sample sizes in available studies highlight the need for larger, high-quality clinical trials to validate these promising outcomes [45]. Future work should utilize multimodal deep learning methods that integrate imaging modalities (e.g., color fundus photography, optical coherence tomography, and angiography), quantitative biomarkers, and clinical data with AI-driven deep learning modalities, enabling earlier detection, precise prognostication, and personalized therapeutic strategies for acute vision loss. The use of ultra-widefield imaging and reinforcement learning-based reasoning frameworks aims to build intelligent, interpretable, and clinically applicable AI systems that support individualized clinical decision-making [46-48]. In summary, future efforts should prioritize early detection, innovative treatments, and interdisciplinary collaboration to improve outcomes and reduce the burden of acute vision loss.

## Conclusions

Acute visual loss remains a time-critical clinical emergency requiring prompt recognition, systematic evaluation, and multidisciplinary collaboration. A structured diagnostic framework, beginning with the assessment of laterality, pain, and onset, enables clinicians to efficiently localize pathology and initiate targeted interventions. The early identification of red flags such as painless monocular loss, visual field defects, or systemic symptoms is crucial to prevent irreversible visual impairment. Advanced imaging modalities, including OCT and fundus fluorescein angiography, alongside emergent neuroimaging, enhance diagnostic accuracy and expedite management decisions. Despite advancements in diagnostics and treatment, delayed presentation, limited specialist access, and fragmented emergency workflows continue to challenge optimal care. Future directions should focus on expanding tele-ophthalmology, integrating artificial intelligence for rapid image interpretation, and strengthening interdisciplinary emergency protocols. Ultimately, adopting a systematic, technology-assisted approach can substantially improve visual and neurological outcomes, reducing the lifelong burden of blindness.
